# Summary and Frequency of Barriers to Adoption of CPOE in the U.S

**DOI:** 10.1007/s10916-015-0198-2

**Published:** 2015-02-01

**Authors:** Clemens Scot Kruse, Kristina Goetz

**Affiliations:** Texas State University, San Marcos, USA

**Keywords:** Computer Physician Order Entry (CPOE), Order entry systems, Medical, Errors, Medical, Delivery of health care, Continuous quality management

## Abstract

Medical errors are common, costly, and can potentially be life threatening to patients. Recent studies have established that Computer Provider Order Entry (CPOE) systems reduce medical errors as well as improve safety, quality, and value of patient care. However, research regarding adoption factors associated with CPOE systems is limited. Therefore, the purpose of this review was to determine adoption factors by identifying the frequency of barriers through the analysis of literature and research. A systematic literature review was conducted from EBSCO Host and Google Scholar. The search criteria focused on publication date, keywords, and peer reviewed articles. The final set established for evaluation was ten articles. The authors summarized each article and then identified common barriers. Throughout the ten articles, 31 barriers were identified; 15 of which were unique. The three most frequent barriers identified were: process changes (23 %), training (13 %), and efficacy (10 %). The results of this review identify to policy makers levers to incentivize to encourage adoption. The results also illustrate to vendors the importance of factors to include in both marketing and development. The leadership of healthcare organizations should be encouraged to see such results and know that their concerns are heard. If policy makers and vendors help healthcare organizations overcome barriers to adoption, the organization has a better chance of successful CPOE implementation. If successfully implemented, a CPOE system can improve the process of care, quality of care, and patient outcomes; all of which address issues of Meaningful Use.

## Introduction

Medical errors are common, costly, and can potentially be life threatening to patients. In 1999, the Institute of Medicine reported that medical error was the cause of at least 44,000, and perhaps up to 98,000, deaths each year in the United States [1]. Approximately 50 % of adverse drug events (ADE) are preventable and can be related to prescription errors [2]. Implementation of a computerized provider order entry (CPOE) system can serve as a potential solution to improve safety, quality, and value of patient care in the United States [1].

The Health Information Management Systems Society (HIMSS) defines CPOE as:

An order entry application specifically designed to assist practitioners in creating and managing medical orders for patient services or medications. This application has special electronic signature, workflow, and rules engine functions that reduce or eliminate medical errors associated with physician ordering processes [3].

In general, CPOE serves as a tool to increase standardization, quality, and efficiency in the delivery of care provided to patients in healthcare organizations.

Benefits associated with implementing a CPOE system is the decrease in adverse drug events (ADEs) and also the decrease in medication errors such as incorrect dosages, incomplete orders, duplicate therapies, drug allergies, abbreviation errors, and illegible orders due to poor handwriting from the providers [1]. CPOE may also reduce medication override dispense rates from automated dispensing cabinets (ADCs), improve the mean turnaround time (TAT) for first-dose medications, increase productivity, and decrease the amount of time from medication dispensing to medication administration. However, the mass array of benefits from implementing a CPOE system has not lead to immediate adoption of CPOE systems by hospitals and providers. Implementation of CPOE systems across hospitals and providers is modest to rare, specifically, less than 15.7 % [4]. Reasons why CPOE adoption has not been widespread can be attributed to the initial start-up costs of CPOE systems, the cost to maintain a CPOE system, and opposition from providers as they believe a CPOE system will decrease their professional autonomy and the ability to practice medicine [5].

Although the adoption of CPOE systems is currently low, the Health Information Policy Council has defined CPOE as “Meaningful Use” technology; therefore, one can predict a rise in the use of CPOE systems in the near future. Previous research suggests that transitioning from written orders to CPOE has resulted anywhere from 17 to 81 % decrease in medication errors with higher reduction rates credited to intensive training consisting of online tutorials, demonstrations, and educational modules, simplicity, transferability, workflow redesign, and the implementation of a decision support system alongside the implementation of CPOE [6]. CPOE systems alongside decision support systems have shown a significant reduction in overdose, under-dose, interval errors, and harmful drug interactions [6]. The main incentive for CPOE usage is the potential it has to remove certain elements of human error, thus improving the process of care [1]. However, implementing a CPOE system alone will not result in guaranteed success; other factors are associated. To date, little research has gone beyond statistical outcomes to analyze adoption factors associated with implementing a successful CPOE system. This study aims to identify such factors by analyzing the frequency of barriers through the analysis of present literature and research. With medication errors occurring anywhere from poor handwriting to inattention to details, data from this study has the potential to be of great use to address adoption factors for a healthcare organization to successfully implement a CPOE system.

## Materials and methods

### Ethical standards

This study did not require the use of human or animal subjects, it is therefore categorized as IRB Exempt, in accordance with 45 CFR 46. This study was performed in accordance with ethical standards of Texas State University, Texas State University School of Health Administration, and the laws of the United States of America.

**Fig. 1:**
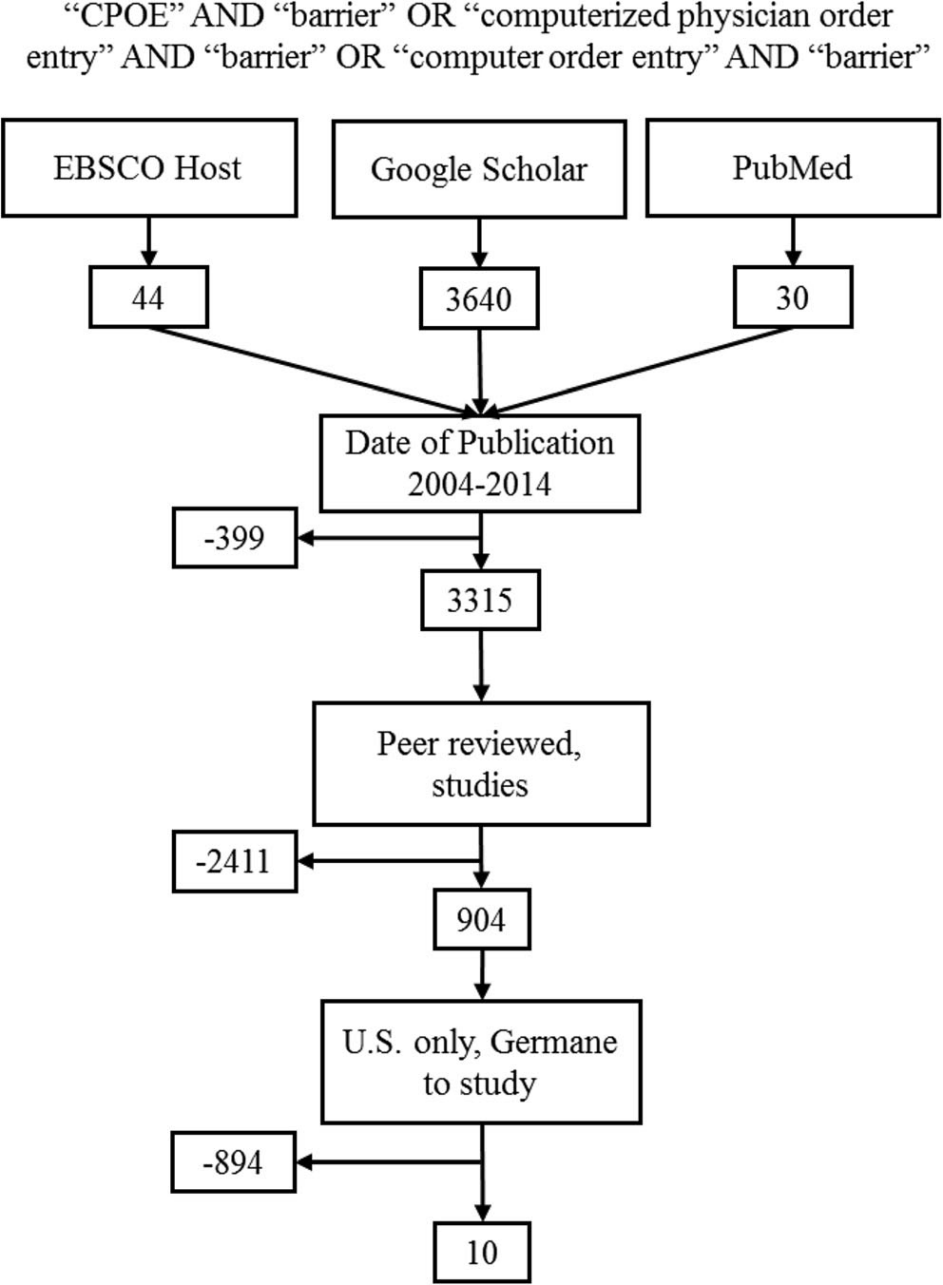
Illustration of the literature review process and rejection criteria

A systematic literature review was conducted on CINAHL (EBSCO host), Google Scholar, and PubMed. Three search phrases were used; each search phrase is separated by the Boolean operator “OR”: “CPOE” AND “barrier” OR “Computerized Physician Order Entry” AND “barrier” OR “Computer Order Entry” AND “barrier.” The literature review process and associated rejection criteria are illustrated in Fig. 1.

Several filters were used in the search engines. The first filter was a date range of 2004–2014. This date range was chosen because of President Bush’s State of the Union Address in 2004 where he placed the adoption of health information technology as one of the top priorities on the national agenda. It was also felt that one decade should be sufficient to capture trends. This filter removed 399 results. The second filter was “peer reviewed journals” (CINAHL) and Google Scholar and “studies” (PubMed). This filter removed 2411 results. From this point, filters in Google Scholar are exhausted, so the results from this search engine became a manual process. The authors briefly examined abstracts from Google Scholar to select those that were germane to our study. In CINAHL and PubMed the additional filter of “U.S. only” was applied, and then the manual process took over. The authors augmented their manpower with the help of another graduate student who was working on a similar topic. The last filter and the manual process eliminated 894 articles, which resulted in the final sample (*n* = 10). With the remaining ten articles, the authors independently reviewed each one, noting the barriers listed in the article. The authors compared notes and chose the barriers that were common between them. Once all barriers from the sample were recorded, the authors assigned a rate of frequency to each different barrier.

## Results

Table 1 identifies barriers associated with each of the ten articles the authors chose for analysis. The articles are listed in no particular order. The numbers in the “Number” column are used for shorthand purposes when identifying a barrier to its corresponding article; these numbers also correspond to the references section. Our team summarized each of the articles and then identified common barriers for the table.

**Table 1:** Study and Barriers Identified

Study	Date	Barriers
Sullins A, Richard A, Manasco K, Phillips M, Gomez T. Which Comes First, CPOE or eMAR? A Retrospective Analysis of Health Information Technology Implementation [7]	2012	• Complexity of the medication • High levels of training required • Process changes • Can take years to realize decrease in error • Efficacy – no universal definition of medication error which makes studies difficult to compare
Radley D, Wasserman M, Olsho L, Shoemaker S, Spranca M, Bradshaw B. Reduction in medication errors in hospitals due to adoption of computerized provider order entry systems [14]	2013	• No universal CPOE solution • Level of adoption / variance • Efficacy – no universal definition of medication error which makes studies difficult to compare
Dow J, Brummond P, Cesarz J, Ludwig B, Rough S. Evaluation of the Impact of Computerized Prescriber Order Entry on Medication Use System Performance at an Academic Medical Center [8]	2012	• High levels of training required • Process changes
Yu F, Menachemi N, Berner E, Allison J, Weissman N, Houston T. Full Implementation of Computerized Physician Order Entry and Medication-Related Quality Outcomes: a study of 3364 hospitals [15]	2009	• No universal CPOE solution • CPOE is complex
Longhurst C, Parast L, Sharek P, et al. Decrease in hospital-wide mortality rate after implementation of a commercially sold computerized physician order entry system [16]	2010	• Cost
Kazley A, Diana M. Hospital Computerized Provider order entry adoption and quality: an examination of the United States [9]	2011	• Cost • Process changes • Resistance of clinicians due to the perception of loss of autonomy
Ballard DJ, Ogola G, Fleming NS, Heck D, Gunderson J, Mehta R, Khetan R, Kerr JD. The Impact of Standardized Order Sets on Quality and Financial Outcomes [10]	2008	• Level of adoption/variance of order sets between organizations • Process changes • High levels of training required • CPOE is complex
Joy A, Davis J, Cardona J. Effect of Computerized Provider Order Entry on Rate of Medication Errors in a Community Hospital Setting [11]	2012	• Process changes • High levels of training required
Galanter W, Falck S, Burns M, Laragh M, Lambert B. Indication-based prescribing prevents wrong-patient medication errors in computerized provider order entry (CPOE) [12]	2013	• Poor user interface • Process changes
Zhan C, Hicks R, Blanchette C, Keyes M, Cousins D. Potential benefits and problems with computerized prescriber order entry: analysis of a voluntary medication error-reporting database [13]	2006	• CPOE eliminates some error, but introduces new error • Poor user interface • Process changes • Legal concerns • Lack of adequate staffing • Successful implementation requires strong leadership endorsement • Efficacy – no universal definition of medication error which makes studies difficult to compare

The analysis of the ten articles revealed a total of 31 barriers present in the implementation of a CPOE system. Of the 31 barriers, 15 barriers were unique. The 15 barriers identified were: CPOE will change the ordering/filling/administration process [7–13], high levels of training are required [7, 8, 10, 11], Efficacy – there is no universal definition of medication error to measure and compare between organizations [7, 13, 14], there is no universal CPOE solution [14, 15], CPOE is complex [10, 15], cost [9, 16], level of adoption within an organization widely varies [10, 14], poor user interface [12, 13], CPOE eliminates some error but introduces new error [13], resistance of the clinicians due to the perception of the loss of autonomy [9], legal concerns [13], lack adequate staffing [13], it can take years to realize the decrease in error associated with a CPOE implementation [7], complexity of the medication [7], successful implementation requires strong leadership endorsement [13].

Table 2 organizes the different barriers by frequency of occurrences with the most frequent listed first. The occurrence rate of the 15 different barriers were: the “process changes” appeared in seven of the ten articles (70 %), and seven of the 31 instances of barriers (23 %); “high levels of training required” appeared in four of the ten articles (40 %), and in four of the 31 instances (13 %); “efficacy” appeared in three of the ten articles (30 %) and three of the 31 instances (10 %); “no universal CPOE solution”, “CPOE is complex”, “cost”, “level of adoption/variance”, and “poor user interface” appeared in three of the ten articles (20 %) and two of the 31 instances (6 %), “CPOE error”, “resistance”, “legal concerns”, “lack of adequate staffing”, ”years for error reduction”, “complexity of medication”, and “leadership” each appeared once out of ten articles (10 %) and once of the 31 instances (3 %).

**Table 2:** Barriers by Number of Occurrences

Number of barriers	Barriers	Frequency	Percent
1	Process changes	7	23 %
2	High levels of training required	4	13 %
3	Efficacy – no universal definition of medication error which makes studies difficult to compare	3	10 %
4	No universal CPOE solution	2	6 %
5	CPOE is complex	2	6 %
6	Cost	2	6 %
7	Level of adoption / variance	2	6 %
8	Poor user interface	2	6 %
9	CPOE eliminates some error, but introduces new error	1	3 %
10	Resistance of clinicians due to the perception of loss of autonomy	1	3 %
11	Legal concerns	1	3 %
12	Lack of adequate staffing	1	3 %
13	Can take years to realize decrease in error	1	3 %
14	Complexity of the medication	1	3 %
15	Successful implementation requires strong leadership endorsement	1	3 %
	Total instances of barriers	31	

## Discussion

Many barriers are associated with the adoption of CPOE. The authors of this study found the most frequent barriers in literature to be: CPOE will change the business process of prescribing, ordering, filling, and administering medication; high levels of training are required for a CPOE implementation; the questionable efficacy of studies due to the differences in defining, recording, and reporting of medication errors. These three errors comprised 46 % of the barriers listed in the literature.

The process changes inherent to a CPOE implementation are not surprising. The implementation of most HIT systems first requires a close examination of the process. There have been some expensive lessons learned from automating a poor process. This barrier could largely be attributed to a fear of change or general resistance to change. As identified by other researchers, a change in process is what is needed the most at the time of discharge, and CPOE serves as the fulcrum of change to this end [17]. Perhaps the solution to this barrier could be found in the second most mentioned barrier: high levels of training required.

The high levels of training required for a successful CPOE implementation is an interesting barrier to be listed in the literature. Studies of implementations most commonly mentioned a lack of adequate training that had been provided, which ranged from 1 to 3 h. It was not clear in the literature whether organizations with the greatest change experienced longer training. It would logically follow, but then it would also be logical that length of training could be indicative of learning styles of the physicians.

The third most often barrier mentioned in the literature was a question of efficacy of CPOE due to the large variation of CPOE solutions, implementations, level of adoption, and definitions of medication error. This is a salient point, and it highlights not only a need for universal definitions, but also a prescribed method of measuring the error. Without such standardization the results of disparate studies could not be compared or combined for a meta-analysis. It is important to note, however, that the use of CPOE for medical orders (to reduce error) is also a salient point of the Meaningful Use criteria.

### Limitations

A limitation of this study was its sample size (*n* = 10). Systematic literature reviews are effective at identifying trends in the area of interest, but a small sample size could threaten the external validity. The methods section of this study enables other researchers to duplicate the study, which maintains a high level of reliability. Other research should broaden the scope of acceptance criteria, specifically publication dates and research engines, to capture a greater sense of the industry, and to increase the external validity.

A second limitation to this study is that the CPOE implementations largely focused on medication errors. The CPOE system, as defined by HIMSS, takes on many more dimensions than just the prescribing, ordering, filling, and administration of medication. This limitation in the literature may largely be due to the ease of measuring medication errors, and the inherent danger in an adverse drug event. It is unlikely that there is sufficient literature on CPOE that focus on other aspects of the medical orders such as rest, physical therapy, ice, elevation, etc.

### Conclusion

The literature demonstrates the ability of CPOE to reduce medical errors, which speaks directly to the pursuit of quality and meeting criteria for the Meaningful Use criteria. However, CPOE systems alone demonstrate limited effectiveness. A successful implementation of CPOE depends on other factors such as gaining strong initial support, strong system champions, deliberate and meaningful design of the system, and adequate staff training. By addressing potential barriers first, a healthcare organization has the potential to gain greater success and support before, during and after implementing a CPOE system. Barriers identified in the literature should be carefully considered when designing factors of adoption associated with implementation of CPOE systems. Policy makers should incentivize the adoption of CPOE through the use of levers that help organizations overcome some of the barriers.
